# Book Reviews

**Published:** 1918-07

**Authors:** 


### BOOK REVIEWS

---

**Books mentioned may be inspected at and ordered through this office. So far as possible, books received in any month will be reviewed in the issue of the second month following. Pamphlets, quarterly and similar periodicals, reports, transactions, etc., will, as a rule, merely be mentioned.**

---

**A Treatise on Clinical Medicine.** By William Hanna Thomson, M. D., L.L.D., formerly Professor of Practice of Medicine and of Diseases of the Nervous System in the New York University Medical College; Ex-President of the New York Academy of Medicine, etc. Second Edition Revised. Octavo volume of 678 pages. Philadelphia and London: W. B. Saunders Company, 1918. Cloth, $5.50 net.

The scope of this volume includes most of the diseases encountered in general practice, and they are classified according to their exciting causes. It begins by discussing the meaning of certain common and important symptoms—"catching cold," pain, emaciation, cough, dyspnea, etc. Then follows a discussion of the remedies used in treatment with practical observation on their limitations and their rational uses. Part II deals with the infections, the "greatest cause of disease and death," prefaced by a discussion of the various infecting organisms. The diseases of special tissues and organs are then taken up in order. The author aims to make his book of practical service to the physician, and this he has accomplished in great measure by eliminating much of the material essential to the text-book and giving us in its place a large amount of information likely to be available to the man who has read the classical authorities and wants something different.

---

**The Medical Clinics of North America.**. Volume 1, Number 5, (The Chicago Number, March, 1918). Octavo of 241 pages, 35 illustrations. Philadelphia and London: W. B. Saunders Company, 1918. Published Bi-Monthly. Price per year: Paper, $10.00; Cloth, $14.00.

Fifteen authors have contributed clinical studies covering a wide range of medical subjects. The temptation to present clinical curiosities has not been allowed to interfere with the practical aims of the editors. The cases are selected with good judgment and the volume is immediately useful to every practitioner.

---

**The Practice of Pediatrics.** By Charles Gilmore Kerley, M. D., Professor of Diseases of Children, New York Polyclinic Medical School and Hospital. Second edition, revised and reset. Octavo of 913 pages, 136 illustrations, Philadelphia and London: W. B. Saunders Company, 1918. Cloth, $6.50 net.

The book begins with the management of the normal child, particularly with reference to diet. The second chapter discusses examination and diagnosis with many useful observations on the management of sick children. The third chapter takes up in detail the diseases of the new-born, and the remainder of the book deals with all the diseases incident to childhood. The last three chapters contain a very complete presentation of the various therapeutic measures applicable to children including a classified list of drugs and dosage for different ages. The author's style is scholarly and sympathetic. He observes that "a well-bred animal, treated from birth to maturity as are many children, would cut a sorry figure in the animal world." Throughout his book he appeals to us to see that the child has a fair chance. In preparing the second edition much material has been added and old chapters have been rewritten where necessary.

---

**Modern Operative Bone Surgery With Special Reference to the Treatment of Fractures.** By Charles George Geiger, M. D. Cloth, 8 vo., XV. 286 pages, with 120 illustrations. Philadelphia, F. A. Davis Co., 1918.

The author devotes his monograph to the consideration of the autogenous bone transplant, which he regards as "THE safe and sound procedure" in osteoplastic surgery. The theme is introduced by an explanation of the histology and physiology of cartilage, periosteum and bone, and the processes by which bone is repaired. In two chapters, well illustrated by roentgenograms, he gives his reason for condemning the use of all foreign material in bone surgery. Over 200 pages of the book are filled with the technical details of the procedures recommended and employed by the author. The Geiger electric motor, for bone work, and many other accessories and devices originated by the author are described in detail with the aid of abundant illustrations. The succeeding chapters deal with all varieties of fractures and abnormal bone conditions to which autogenous bone transplants can be applied. The book is unique and indispensable to the surgeon who works in this precise and difficult specialty.

---

**New York State Report** of the State Commissioner of Highways, 1916. A splendid map accompanies this book.

